# In Vivo Induction of Functionally Suppressive Induced Regulatory T Cells from CD4^+^CD25^-^ T Cells Using an Hsp70 Peptide

**DOI:** 10.1371/journal.pone.0128373

**Published:** 2015-06-24

**Authors:** Martijn J. C. van Herwijnen, Ruurd van der Zee, Willem van Eden, Femke Broere

**Affiliations:** Department of Infectious Diseases and Immunology, Utrecht University, Utrecht, the Netherlands; Université Paris Descartes, FRANCE

## Abstract

Therapeutic peptides that target antigen-specific regulatory T cells (Tregs) can suppress experimental autoimmune diseases. The heat shock protein (Hsp) 70, with its expression elevated in inflamed tissue, is a suitable candidate antigen because administration of both bacterial and mouse Hsp70 peptides has been shown to induce strong immune responses and to reduce inflammation via the activation or induction of Hsp specific Tregs. Although two subsets of Tregs exist, little is known about which subset of Tregs are activated by Hsp70 epitopes. Therefore, we set out to determine whether natural nTregs (derived from the thymus), or induced iTregs (formed in the periphery from CD4^+^CD25^-^ naïve T cells) were targeted after Hsp70-peptide immunization. We immunized mice with the previously identified Hsp70 T cell epitope B29 and investigated the formation of functional iTregs by using an *in vitro* suppression assay and adoptive transfer therapy in mice with experimental arthritis. To study the *in vivo* induction of Tregs after peptide immunization, we depleted CD25^+^ cells prior to immunization, allowing the *in vivo* formation of Tregs from CD4^+^CD25^-^ precursors. This approach allowed us to study *in vivo* B29-induced Tregs and to compare these cells with Tregs from non-depleted immunized mice. Our results show that using this approach, immunization induced CD4^+^CD25^+^ T cells in the periphery, and that these cells were suppressive *in vitro*. Additionally, adoptive transfer of B29-specific iTregs suppressed disease in a mouse model of arthritis. This study shows that immunization of mice with Hsp70 epitope B29 induces functionally suppressive iTregs from CD4^+^CD25^-^ T cells.

## Introduction

Several mechanisms of tolerance are supposed to prevent autoimmunity, excessive inflammatory responses, and to maintain immune homeostasis. CD4^+^CD25^+^ regulatory T cells (Tregs) are specialized CD4^+^ T helper cells that are of great significance to central tolerance [1]. Two main subsets of Tregs exist: natural Tregs (nTregs) or induced Tregs (iTregs) [2]. nTregs originate from the thymus as mature Tregs and are mostly directed against self-antigens [2], while iTregs are formed in the periphery from naïve CD4^+^ T cells in response to mostly foreign antigens [3, 4]. Both subsets have been shown to suppress a variety of immune responses. However, the relative contribution of each subset is still largely unknown and might depend on the specific immunological context [2].

Targeting of antigen-specific Tregs with immunomodulatory epitopes can be used to suppress inflammatory immune responses in animal models of autoimmune diseases [5–8]. Choosing a suitable candidate epitope can be difficult for diseases for which the disease-inducing antigen is unknown, which is the case for rheumatoid arthritis. Therefore, we propose to use antigens that are constitutively expressed, and preferentially upregulated during inflammatory disease. One such antigen is heat shock protein (Hsp) 70, an evolutionary conserved protein that is expressed and upregulated in the inflamed synovium [9] and of which bacterial homologs have been shown to induce immune responses upon immunization [10].

Previously, we have shown that administration of Hsp70 or Hsp70-derived peptides suppresses experimental arthritis via the activation of CD4^+^CD25^+^FoxP3^+^ Tregs [10, 11]. Immunization of mice with the mycobacterial Hsp70 epitope B29 generated CD4^+^CD25^+^FoxP3^+^ T cells that were cross reactive with mouse Hsp70 peptides and able to suppress established arthritis upon transfer, whereas such cells from animals immunized with control antigen pOVA were not able to suppress disease. These results suggest that B29-specific Tregs need to be activated *in vivo* by locally presented mouse B29 homologs [10].

However, it is unknown whether the administration of B29 peptide converts naïve T cells into B29-specific iTregs, or that peptide administration expands already existing B29-specific nTregs. It is important to establish the contribution of Treg subsets in suppression of disease after peptide administration in order to fine-tune peptide based therapies to optimally target Tregs in future therapies. Therefore, we set up a protocol to induce Tregs *in vivo* by first removing CD25^+^ Tregs with anti-CD25 depleting antibody, leaving CD4^+^CD25^-^ naïve T cells untouched, followed by subsequent B29 peptide immunization. We hypothesized that if B29-specific naïve T cells exist, they become iTregs after encounter with B29.

Here, we show that immunization with the Hsp70 peptide B29 after depletion of CD25^+^ cells, induced CD4^+^CD25^+^ cells that were equally suppressive *in vitro* and *in vivo* as CD4^+^CD25^+^ cells from B29 immunized mice without prior depletion. This suggests that B29-immunization can induce antigen-specific iTregs from naïve CD4^+^CD25- T cells.

## Materials and Methods

### Mice and peptides

Female Balb/c mice were purchased from Charles River and for peptide immunization 8–12 week old mice were used. For proteoglycan induced arthritis (PGIA) experiments, retired breeders were used. Animals were kept under standard conditions at the animal facility and all experiments were approved by the Animal Experiment Committee of Utrecht University. Peptides were purchased from GenScript Corporation (B29, mB29a, mB29b and pOVA _323–339_; for details see [10]).

### Immunization and depletion of CD25^+^ cells for cell isolation, restimulation and flow cytometry

**Fig 1:**
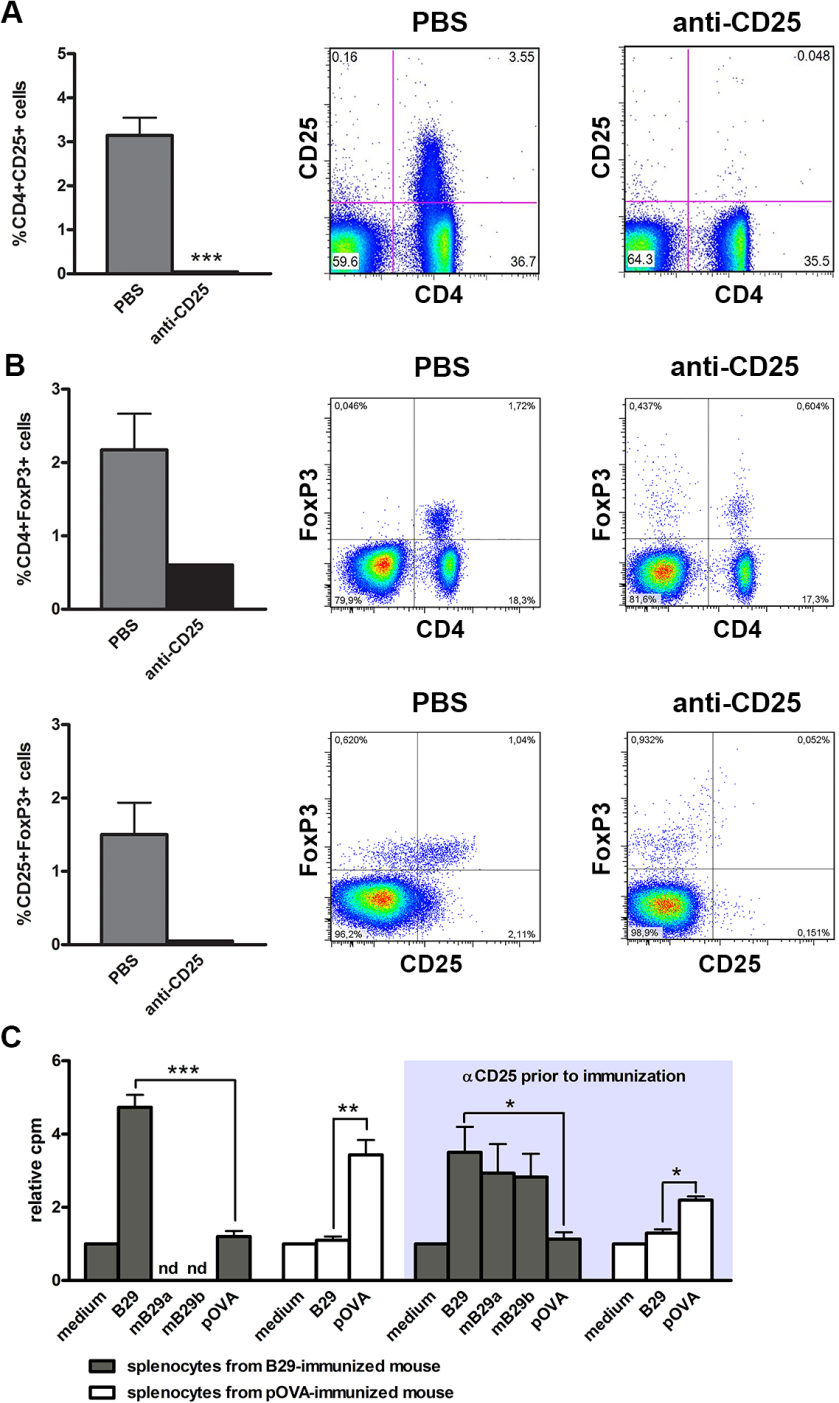
B29-specific T cell proliferation in mice immunized with B29 after CD25^+^ T cell depletion. Mice were injected with anti-CD25 depleting antibody PC61 or with PBS as a control. 7 days after depletion of CD25^**+**^ cells, the mean percentage (± s.e.m.) of CD25^**+**^ cells (A) or FoxP3^**+**^ cells (B) was determined in total peripheral blood directly prior to immunization of n = 2–6 (A) or n = 1–3 (B) animals per group. Data of figure A are representative of 3 independent experiments. (C) 7 days after administration of anti-CD25 antibody (depicted as αCD25) or PBS, mice were immunized with Hsp70 peptide B29, or control peptide pOVA, and 10 days later splenocytes were restimulated with B29, mouse homologs mB29a or mB29b, or control peptide pOVA. Results are expressed as the mean relative cpm (cpm peptide / cpm medium only ± s.e.m.) obtained from of 3–4 animals per condition and are representative of 3 independent experiments. Background cpm values of the negative controls were as follows (all medium controls from left to right): medium control of B29-immunized mouse 1004 cpm; medium control of pOVA immunized mouse 738 cpm; medium control of B29-immuinized mouse + αCD25 prior to immunization 4088 cpm; medium control pOVA immunized mouse + αCD25 prior to immunization 2055. nd: not determined. P values are from an unpaired two-tailed Student t test in which the PBS group was compared to the anti-CD25 antibody treated group (A), or in which Hsp70 peptide (B29, mB29a, or mB29b) stimulation was compared to pOVA stimulation (B). *P < 0.05; **P <0.01; ***P < 0.001.

**Fig 2:**
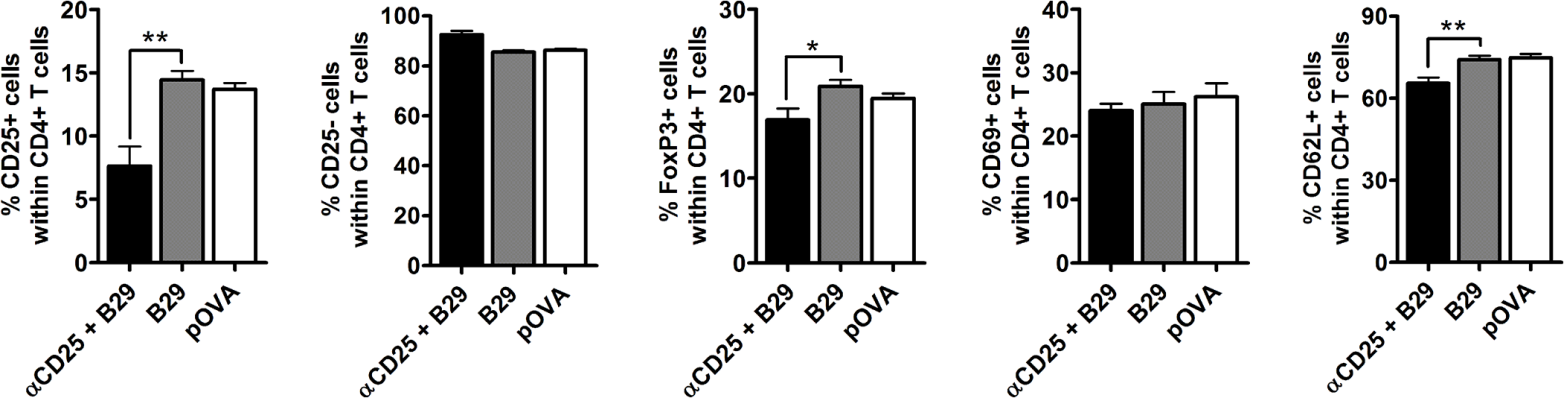
Induction of CD4^+^CD25^+^ and CD4^+^FoxP3^+^ cells after peptide immunization in mice prior depleted from CD25^+^ cells. Mice were injected with anti-CD25 antibody or with PBS only. 7 days later, mice depleted from CD25^**+**^ cells were immunized with B29 (depicted as αCD25+B29). Mice that received PBS were immunized with B29 or pOVA (depicted as B29 or pOVA). 10 days after peptide immunization, mice were sacrificed and splenic CD4^**+**^ T cells were assessed for Treg markers and activation markers by flow cytometry. The results depicted are the mean percentages (± s.e.m.) of CD25^**+**^, CD25^**-**^, FoxP3^**+**^, CD69^**+**^ and CD62L^**+**^ cells within the CD4+ T cell population of the spleen. Data are the mean of 8 animals per group. P values are from an unpaired two-tailed Student t-test in which the αCD25 + B29 group was compared to B29 group. * P < 0.05, ** P < 0.01.

**Fig 3:**
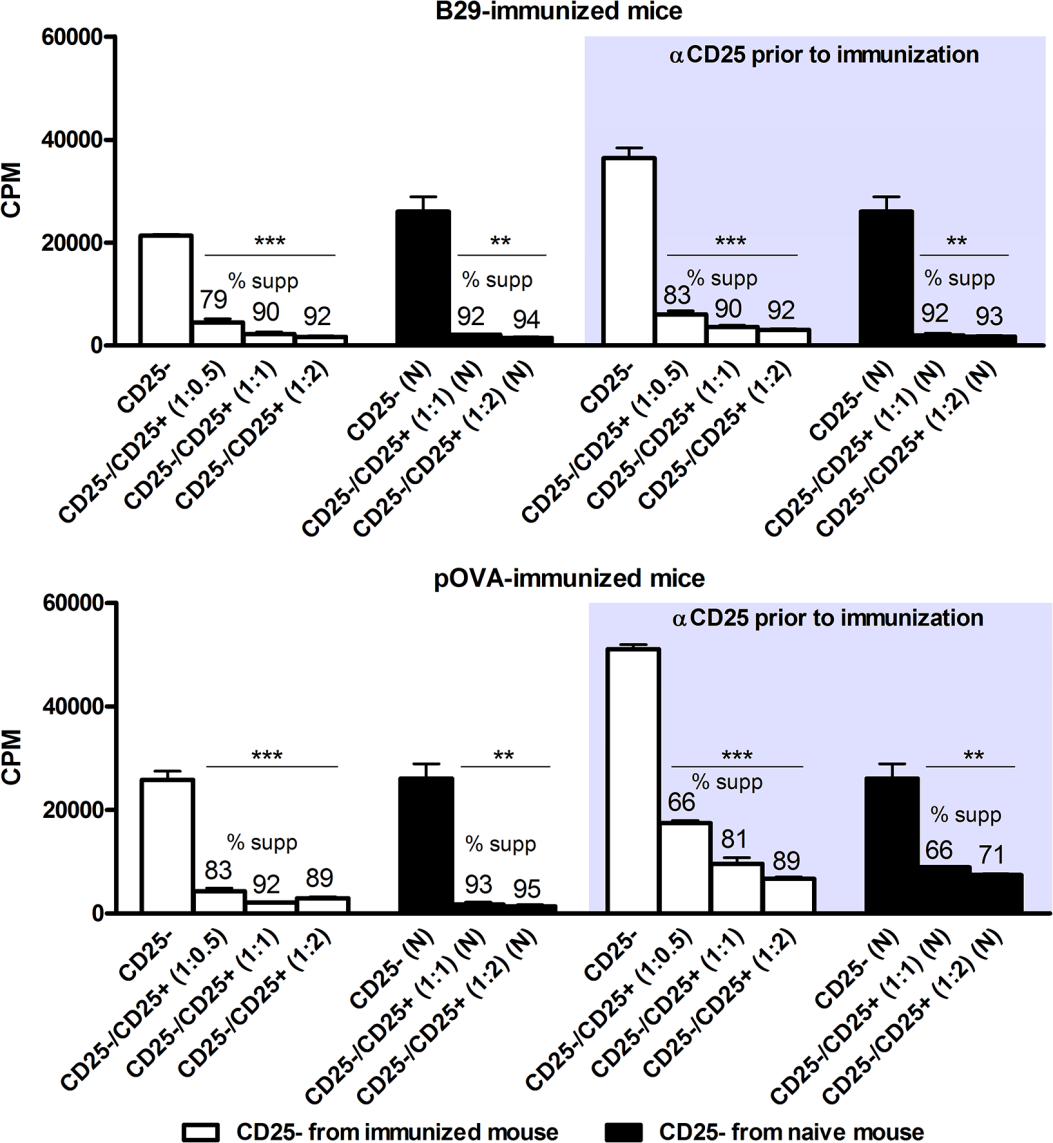
B29 induced Tregs are suppressive in vitro. Mice were either injected with anti-CD25 antibody to deplete CD25^**+**^ cells, or with PBS as a control. 7 days after injection, mice (n = 3 per treatment) were immunized with either B29 (upper graph) or pOVA (lower graph). 10 days later autologous CD4^**+**^CD25^**-**^ responder cells (white bars) and CD4^**+**^CD25^**+**^ cells were isolated and pooled for co-culture in various ratios in the presence of anti-CD3 antibody to activate the cells. As a control, also CD4^**+**^CD25^**-**^ responder cells from naïve (N) donors (black bars) were used to test the suppressive capacity of B29-induced Tregs or pOVA-induced Tregs on the same population of responder T cells. ^**3**^H-thymidine incorporation was determined and cpm data are shown as the mean of triplicate samples (± s.e.m.). % supp. is the proliferative response of responder T cells cultures alone, compared to responder T cells co-cultured with Tregs. Data shown are representative for two independent experiments. P values are from an unpaired two-tailed Student t-test in which cpm from CD4^**+**^CD25^**-**^ cells were compared to cpm from CD4^**+**^CD25^**+**^ cells. ** P < 0.01, *** P < 0.001.

**Fig 4:**
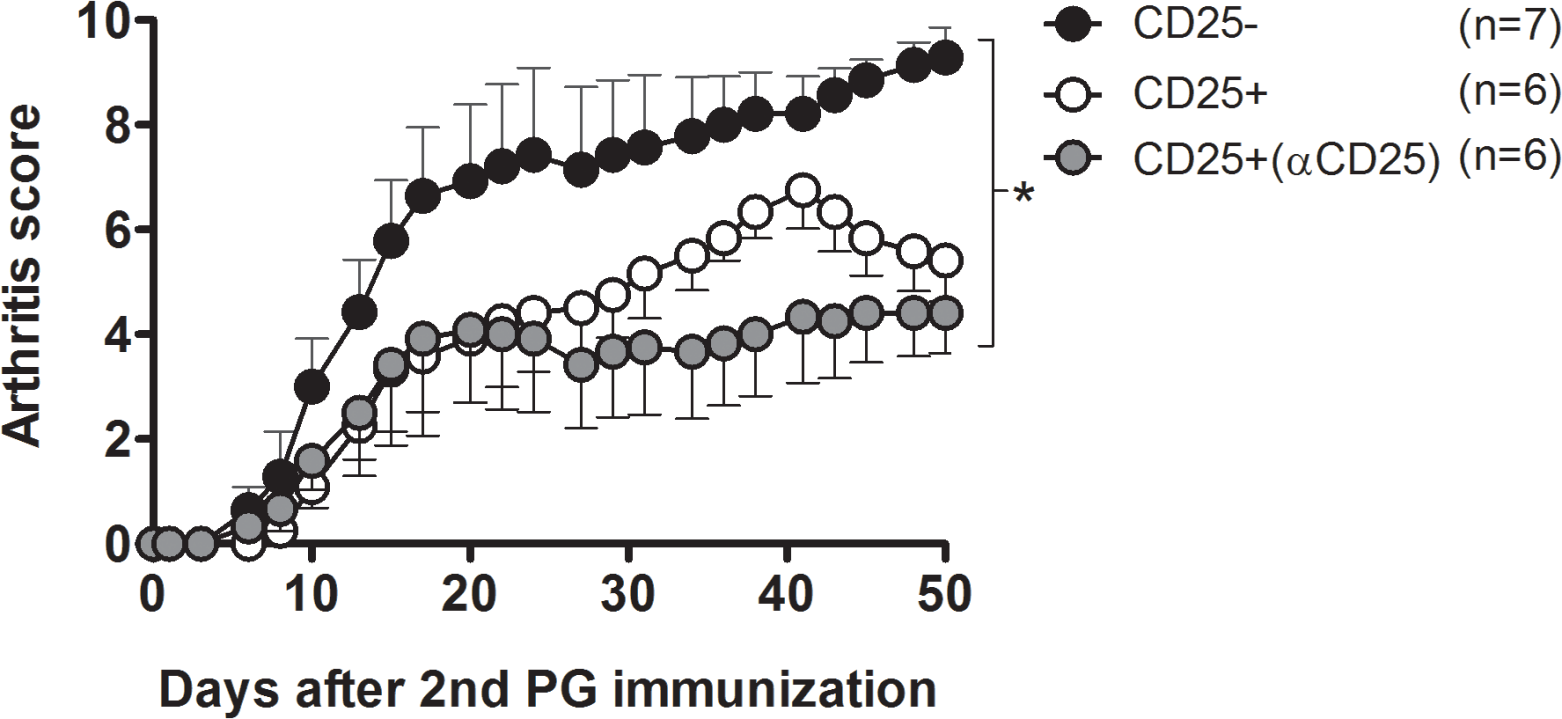
Adoptive transfer of B29-induced Tregs reduces inflammation in a mouse model of rheumatoid arthritis. Mean arthritis scores of recipient mice after adoptive transfer of CD4^**+**^CD25^**-**^ cells or CD4^**+**^CD25^**+**^ cells from B29-immunized donors (injected with PBS 7 days prior to B29 immunization), or mice receiving CD4^**+**^CD25^**+**^ cells from B29-immunized donors injected with anti-CD25 antibody 7 days prior to immunization (depicted as CD25+ αCD25). Recipient animals (n = 6–7 mice per group) received 3x10^**5**^ cells i.p. one day prior to the second PG immunization. Clinical scores were assessed over time and are depicted as the mean of the group (± s.e.m.). Data shown are representative for 2 experiments. P values are from a two-way ANOVA (all time points) followed by Bonferroni post hoc comparison. *P < 0.05.

Mice were immunized with 100 μg peptide (mycobacterium Hsp70 peptide B29, or pOVA) with 2 mg Dimethyldioctadecylammonium bromide (DDA) in 200 μl PBS via i.p. plus s.c. injection. 10 days later, mice were sacrificed and splenocytes were isolated as described previously [10]. For restimulation (Fig 1B) and flow cytometry (Fig 2), splenocytes from individual mice were analyzed separately. For *in vitro* suppression assays (Fig 3) and adoptive transfer experiments (Fig 4), spleens were pooled per group and CD4^+^ cells were isolated using Dynal bead isolation (Invitrogen) by negatively selecting CD4^+^ T cells, followed by FACS sort (influx, BD) to isolate CD4^+^CD25^-^ or CD4^+^CD25^+^ with purities up to 96%. For depletion of CD25^+^ cells, mice were given 400 μg anti-CD25 antibody (PC61, produced in house from hybridoma ATCC PC61 and purified from supernatants) in 200 μl PBS i.p. Immunization with peptide followed 7 days after administration of anti-CD25 antibody, the control group received 100 μl PBS i.p 7 days prior to peptide immunization. The timeline for depletion and subsequent immunization was: t = 0 administration of anti-CD25 antibody or PBS, t = 7 immunization with B29 or pOVA, t = 17 sacrifice mice and isolation spleen.

### Flow cytometry and antibodies

Flow cytometry was performed with CantoII (BD) with monoclonal antibodies CD4-FITC (RM4-5, eBioscience), CD4-PerCP (RM4-5, BD Bioscience), CD25-APC (PC61, BD Bioscience), CD62L-FITC (MEL-14, BD Bioscience), CD69-FITC (H1.2F3, BD Bioscience) and FoxP3-PE (FJK-16, eBioscience). For Fig 1A, whole blood cells were obtained via the submandibular vein 7 days after administration of anti-CD25 antibody (which would be the time point for peptide immunization). Red blood cells were lysed with ACK (Ammonium-Chloride-Potassium) buffer. Remaining cells were stained for CD25 and the percentage of CD25^+^ cells was determined for the entire population of blood cells. For Fig 2, single cell suspensions of splenocytes were stained for CD4 in combination with CD25, FoxP3, CD69, or CD62L.

### Restimulation and suppression assay

For restimulation, splenocytes were harvested from mice immunized as described above and cultured in 96-wells flat bottom plates in triplicate wells in a concentration of 2x10^5^ cells/well. Cells were restimulated with 20 μg/ml peptide (B29; the mouse homologs of B29: mB29a or mB29b; or control peptide pOVA) for 72h, and ^3^H-thymidine was added for another 18h. Relative cpm was measured by dividing the counts per minute (cpm) of the stimulated conditions by the medium (unstimulated) cpm. For the *in vitro* suppression assay, CD4^+^CD25^-^ responder T cells (1x10^5^) and CD4^+^CD25^+^ T cells (0.5x10^5^, 1x10^5^, or 2x10^5^) were purified by FACS sort as described above and cells were co-cultured in triplicate wells with irradiated splenocytes (2x10^5^) as APC in 96 wells flat bottom plates. Cells were stimulated with 5 μg/ml soluble anti-CD3 antibody (clone 145-2C11) for 72h. ^3^H-thymidine was added for an additional 18h and the percentage suppression (depicted as % supp in Fig 3) was calculated from cpm values obtained from stimulated responder T cells only.

### Proteoglycan Induced Arthritis (PGIA) and adoptive transfer

Retired breeders were immunized twice with an interval of 21 days with 250–300 μg human proteoglycan (PG) protein with 2 mg DDA given in 200 μl PBS via i.p. injection, as was previously described [12]. Mice were randomly grouped and arthritis was scored in a blinded fashion using a visual scoring system based on swelling and redness as described previously [12]. For adoptive transfer, 3x10^5^ donor cells were given i.v. in 100 μl PBS to recipient mice one day prior to the second PG immunization. Three groups of recipients got donor cells: 1) CD4^+^CD25^-^ cells isolated 10 days after immunization with B29 peptide. 2) CD4^+^CD25^+^ cells from the same B29-immunized donors as group 1. 3) CD4^+^CD25^+^ cells isolated 10 days after immunization from B29-immunized donors, which were injected with anti-CD25 antibody 7 days prior to immunization.

### Statistical analysis

Data is shown as mean ± standard error of mean (s.e.m.). Statistics were done using Prism 4 (Graphpad Software Inc.). Comparisons between two groups were done with the Student’s *t*-test (unpaired and two-tailed). Multigroup comparisons were done by using a two-way ANOVA followed by Bonferroni post hoc comparison. P values less than 0.05 with a 95% confidence interval were considered significant, with * P <0.05, ** P <0.01, *** P <0.001.

## Results

### B29-specific T cell proliferation in mice immunized with B29 after CD25^+^ T cell depletion

In this study, we wanted to elucidate the role of the iTreg subset after B29 immunization and test B29-specific iTreg mediated suppression in autoimmunity.

We have set up an experimental procedure to characterize the induction and suppressive capacity of B29-specific iTregs by depletion of nTregs prior to immunization with B29. For this, we depleted CD25^+^ cells and FoxP3^+^ cells (including CD4^+^CD25^+^FoxP3^+^ nTregs) *in vivo* with anti-CD25 antibody PC61 (Fig 1A and Fig 1B). We hypothesized that subsequent peptide immunization after depletion of nTregs results in the formation of antigen-specific iTregs (and effector T cells) from CD4^+^CD25^-^ precursors while minimizing the expansion of antigen-specific nTregs. To test this, we first investigated whether B29-immunization after depletion of all CD25^+^ cells resulted in antigen-specific T cell responses. *In vitro* restimulation of splenocytes from immunized mice showed that depletion of CD25^+^ cells (including Tregs) followed by peptide immunization resulted in T cell proliferation that was peptide specific (Fig 1C, right panel). In addition to B29 mediated proliferation, cross-reactive responses to the previously identified mouse homologs mB29a and mB29b were observed in mice immunized with B29. Depletion of CD25^+^ cells prior to immunization resulted in higher counts for all restimulation conditions (data not shown), indicating that the absence of CD25^+^ Tregs during the immunization period resulted in increased T cell priming. However, since the ^3^H-thymidine incorporation of both stimulated and unstimulated splenocytes increased relatively with the same magnitude (see legend Fig 1C), the relative cpm was more or less the same as the peptide-specific responses without CD25 depletion (Fig 1C, left panel). These results show that depletion of CD25^+^ cells prior to peptide immunization does not affect the induction of antigen-specific T cells.

### Induction of CD4^+^CD25^+^ and CD4^+^FoxP3^+^ cells after peptide immunization in mice prior depleted from CD25^+^ cells

Next, we determined the induction and phenotype of splenic Tregs 10 days after immunization with B29 in mice that were prior depleted from CD25^+^ cells and compared these with Tregs from undepleted mice immunized with B29 or control peptide pOVA. Depletion of CD25^+^ cells 7 days prior to immunization with B29 resulted in a significant reduction of CD25^+^ cells in the CD4^+^ T cell population (Fig 2). Anti-CD25 antibody administration prior to immunization resulted in less prominent reduction of FoxP3^+^ (Treg) cells and CD62L^+^ (naïve) cells, while the percentages of CD25^-^ and CD69^+^ cells were not significantly affected. Since CD25^+^ cells were almost completely lacking 7 days after anti-CD25 antibody administration (which is the time of subsequent peptide immunization: Fig 1A), these results indicate the induction of CD25^+^ and FoxP3^+^ cells after subsequent peptide immunization, in the following 10 days.

### B29-induced CD4^+^CD25^+^ T cells are suppressive *in vitro*

We hypothesized that B29-peptide immunization converted naïve CD25^-^ cells into suppressive CD4^+^CD25^+^ cells. Therefore, we first determined whether newly formed CD4^+^CD25^+^ cells from B29-immunized or pOVA-immunized mice pre-treated with anti-CD25 antibody, were suppressive *in vitro*. For this, CD4^+^CD25^+^ T cells were isolated from splenocytes of immunized mice, either pre-treated with anti-CD25 antibody or not. As a readout of suppression, anti-CD3 induced proliferation of autologous CD4^+^CD25^-^ responder T cells (white bars) in the presence of different numbers of CD4^+^CD25^+^ T cells was determined (Fig 3). Since depletion of CD25^+^ cells prior to peptide immunization resulted in higher proliferation of activated responder T cells cultured alone (depicted as CD25^-^), we also included CD4^+^CD25^-^ responder cells from naïve mice (black bars) to compare the suppressive capacity of CD4^+^CD25^+^ T cells between depleted and non-depleted mice. The data show that CD4^+^CD25^+^ cells from B29-immunized mice were equally suppressive, irrespective of prior depletion of CD25^+^ cells (Fig 3, upper graph). This suggests that suppressive CD4^+^CD25^+^ cells from B29-immunized mice pre-treated with anti-CD25 antibody are formed *de novo* from CD25^-^ cells, although we cannot exclude the formation of Tregs specific for other antigens than B29. Immunizing depleted mice with pOVA also resulted in suppressive CD4^+^CD25^+^ cells, although these pOVA-induced cells were less suppressive than B29-induced CD4^+^CD25^+^ cells (Fig 3, lower graph) indicating that in the pOVA-immunized mice the suppressive activity was not fully restored after the preceding depletion, and thus that immunization with B29 resulted in the *de novo* formation of potent suppressor cells.

### Adoptive transfer of B29-induced Tregs reduces inflammation in a mouse model of rheumatoid arthritis

To test whether B29-induced Tregs were capable of suppressing inflammation *in vivo*, we immunized two groups of donor mice with B29, either i.p. pre-treated with anti-CD25 antibody or with PBS only. Transferring CD4^+^CD25^+^ T cells from B29-immunized donors from both, differently pre-treated groups, resulted in a similar suppression of clinical symptoms of arthritis as compared with the transfer of CD4^+^CD25^-^ control cells (Fig 4). These results are in line with the *in vitro* suppression data (Fig 3), indicating that B29-immunization one week after depletion of CD25^+^ cells induced new CD4^+^CD25^+^ T cells that are suppressive. Thus both *in vitro* and *in vivo*, these B29 induced CD4^+^CD25^+^ T cells from depleted donors were equally suppressive as the CD4^+^CD25^+^ T cells from B29 immunized, non-depleted donors. This indicates that B29 immunization in non-depleted donors led to the induction of iTregs, rather than activation of preexisting nTregs.

## Discussion and Conclusion

Successful use of Hsp peptides for the inhibition of inflammation has been shown both in animal models [11, 13] and in clinical trials with autoimmune patients [14, 15]. The immunogenic nature of Hsp [16, 17] as well as their upregulation under inflammatory conditions [9, 18, 19] make these proteins suitable candidate antigens for the suppression of autoimmune diseases even when disease-causing antigens are unknown, as is the case for rheumatoid arthritis. The peptide specific suppression after Hsp administration comes from Tregs responsive to the Hsp peptides [10]. Activating antigen-specific Tregs seems crucial for optimal suppression, since antigen-specific Tregs were shown to be superior over polyclonal Tregs upon transfer [20, 21]. Recently, we showed that immunization or intranasal administration of Hsp70 peptide B29 activated Hsp-specific Tregs that suppressed experimental arthritis upon adoptive transfer. Hsp-specific Tregs were suppressive in low numbers, especially when selected on lymphocyte activation gene (LAG)-3 expression. The transferred cells remained present in lymphoid tissues up to 3 months after injection where they had an activated phenotype [10]. Thus, although the presence and suppressive activity of Hsp-specific Tregs has been shown, nothing is known about the type of subset of Treg that is activated after Hsp administration.

The Treg population can be divided into two subsets: nTregs derived from the thymus (mostly self-specific) [22] and iTregs (mostly specific against foreign peptides) [23] that are formed in the periphery from naïve T cells [23]. It is still largely unknown what the contribution of the individual subset of nTregs or iTregs in immune modulation is. Some studies have addressed the contributions of both subsets to immune tolerance. For instance, adoptively transferred nTregs isolated from thymus can partially suppress autoimmunity in Foxp3 deficient mice, although complete rescue from disease only takes place in het presence of *in vitro* generated iTregs [24]. The authors suggested that since both subsets have different TCR repertoires they are complementary, rather than redundant. Since Hsp peptides can either originate from self Hsp [25–28], or from bacterial Hsp in the gut [29, 30] and/or infections [31], this suggests that Hsp-specific Tregs can be present both in the induced and in the natural subset of Tregs. Therefore, we set out to investigate whether Hsp-specific iTregs are able to suppress experimental arthritis.

In this study we show that administration of anti-CD25 antibody PC61 resulted in the absence of CD4^+^CD25^+^ cells at day 7 day after injection (Fig 1A) and gave a reduction of CD4^+^FoxP3^+^ T cells after injection (Fig 1B). Additionally, in the Balb/c mice used in our studies at least 90% of the CD25^+^ cells express FoxP3 [10], and it has been shown in previous studies that the anti-CD25 antibody PC61 depletes FoxP3^+^ cells [32, 33]. Subsequent immunization with the Hsp70 peptide B29 gave antigen responsive splenocytes that were responsive to the B29 peptide, or its homologs (Fig 1C). In addition, immunization of CD25 depleted mice resulted in the presence of CD4^+^CD25^+^ T cells, although less than in undepleted mice that had been immunized (Fig 2). The CD4^+^CD25^+^ T cells present after immunization were tested for their suppressive function *in vitro* (Fig 3). Depletion of CD25^+^ cells prior to immunization and *in vitro* anti-CD3 stimulation resulted in higher proliferation of CD4^+^CD25^-^ cells, indicating that CD25^+^ T cells give a basal immune suppression. There was no difference in the suppressive capacity of CD4^+^CD25^+^ cells from non-CD25 depleted B29-immunized mice compared to CD4^+^CD25^+^ from mice that were pre-treated with anti-CD25 before B29 immunization. This shows that B29-immunization in CD25 depleted mice results in the *de novo* formation of suppressive CD4^+^CD25^+^ T cells. On the other hand, the same approach for pOVA did not result in equally suppressive CD4^+^CD25^+^ T cells indicating that in the pOVA-immunized mice the suppressive activity was not fully restored after the previous depletion. This underlines our previous finding that the B29 epitope induces a regulatory response after immunization [10]. Next to the *in vitro* suppressive capacity, B29-induced CD4^+^CD25^+^ T cells were tested for their *in vivo* suppression in a mouse model for experimental arthritis. Upon adoptive transfer, the B29-induced CD4^+^CD25^+^ cells suppressed experimental arthritis (Fig 4), due to cross recognition of mouse homologs of B29. These results are in line with previous work that reported that *in vitro* induced Tregs suppressed disease in arthritic animal upon transfer [10, 34]. However, in the latter studies CD4^+^CD25^+^ cells were induced *in vitro* from naïve cells, whereas in the present study we employed *in vivo* induced Tregs. For therapeutic purposes, it would be interesting to amplify the conversion of iTregs from naïve T cells through rapamycin [35, 36], IL-2 [37, 38] or anti-CD3 [39]. In the case of therapies in RA, we would suggest to use anti-inflammatory drugs, such as anti-TNFα for instance, to allow for a window of opportunity for the formation of iTregs before peptide administration.

Using the method described in this paper, we were able to convert CD4^+^CD25^-^ cells into antigen-specific CD4^+^CD25^+^ Tregs *in vivo* through immunization with Hsp70 peptide B29. However, we cannot fully exclude that, partially, new CD25^+^ cells are formed in the 10 days after immunization that are not B29-specific, or that are CD25^+^ nTregs. We expect that the bulk of newly formed CD25^+^ cells is most likely due to proliferation caused by immune activation after immunization and that any contaminating cell will have little influence on the effects observed. There is debate whether specific phenotypic markers for Treg subsets exist to distinguish nTregs from iTregs whenever origin of the cells is unknown, for instance in *ex vivo* analysis. As a marker for nTregs, Helios was considered until later it was shown that this transcription factor identifies the activation status of Tregs, irrespectively of their origin [40]. Therefore screening for nTreg or iTregs markers can not be used to discriminate the two populations in our model. Currently, the methylation status of FoxP3 is perhaps the best marker to identify Tregs subsets [41–43]. The CD4^+^CD25^+^ cells formed in B29-immunized mice depleted from CD25^+^ cells expressed FoxP3 (Fig 2), thus identifying the methylation status of FoxP3 in these cells could provide additional information about to what extent these cells are iTregs.

In conclusion, in this study we show the induction of CD4^+^CD25^+^ Tregs via immunization with the Hsp70 epitope B29 in mice depleted of CD25^+^ cells. We show that *de novo* induced Tregs after Hsp70 peptide immunization are suppressive *in vitro* and suppress experimental arthritis upon adoptive transfer to the same extent as the total Tregs population of immunized donors. This indicates that the suppression seen after adoptive transfer therapy was due to iTregs.
